# The Investigation of Uric Acid in Salivary Tartar in the Course of Pyorrhea Alveolaris (Artaro-Dental Infectious Gingivitis)

**Published:** 1897-02

**Authors:** 


ARTICLE IV.

### THE INVESTIGATION OF URIC ACID IN SALIVARY TARTAR IN THE COURSE OF PYORRHEA ALVEOLARIS (ARTARO-DENTAL INFECTIOUS GINGIVITIS).

BY DR. GALIPPE.

Like all grave maladies whose etiology is imperfectly understood and whose treatment is slow and difficult, pyorrhea alveolaris has received a considerable number of appellations, and its etiology has been attributed to the most diverse pathological influences.

I was not a little surprised on reading, in 1894, in an American selection, that the etiology of pyorrhea alveolaris must be always attributed to gout. The author of this assertion based himself upon the invariable presence of uric acid or of urates in salivary tartar, as well as in the deposits forming incrustation at the extremity of teeth which have dropped out, or have been extracted in the course of this disease.

I have made it my task to examine this assertion, with the help of two of my collaborators, MM. Brun et Gaillard Starting with this fact; that if uric acid really exists in salivary tartar, or at the extremities of the roots, it ought to be found there in a relatively feeble quantity, our researches were carried out with the most minute care and the greatest precaution. The American author does not say by what means he has been able to verify the presence of the uric acid. We have not, perhaps, followed the same path in our investigations as he took. We have adopted the most commonsense classic methods, those which are ordinarily followed when the presence of uric acid has to be determined in concretions.

These methods are of two kinds: I. If we treat the concretions inclosing the finely pulverized uric acid by azotic acid, then evaporate the excess of uric acid, and add to the residue a drop of diluted liquid ammonia, we obtain immediately the rose-purple coloration of murexide. II. If we treat the same concretions by an alkaline solution of potassium, the uric acid becomes dissolved, giving place to a formation of soluble urate of potassium. To the filtered liquid is added hydrochloric acid, which produces a precipitate of uric acid, easily to be recognized on examination with the microscope.

1. With these methods we first made some trials upon saliva; our results were negative. 2. Upon ten samples of fresh tartar we also obtained negative results, 3. Upon forty teeth, studied separately, and chosen from patients affected with pyorrhea alveolaris, the tartar was first detached and analyzed, thereupon the extremities of the roots were sawed off and examined after pulverization. All the results were negative.

The salivary tartar taken from several series of teeth (9, 6 and 4) did not reveal the presence of uric acid; the combined roots of these same series did not afford better results.

In the face of these persistent negative results we asked ourselves, after the first three or four trials, if the presence of the organic and mineral matter in the salivary tartar did not prevent the reactions. We therefore instituted experiments of control, which were subsequently made after each examination of tartar or of root. 1. To saliva we added extremely weak quantities of uric acid, and these infinitesimal proportions gave us very good reactions. 2. The same very feeble quantities of uric acid were added to the fresh salivary tartar, also to the dry salivary tartar, and likewise to the finely pulverized extremities of the roots of the teeth. In all the cases we were able to demonstrate clearly the presence of the added uric acid.

We therefore believe that we are right in concluding: 1. That the experiments were made under conditions and with sufficient care to reveal the presence of even very small quantities of uric acid. 2. That, if we have not demonstrated the presence of this acid, either in the saliva or in the fresh and dry tartar, or in the extremities of the roots of the teeth, it is because there was not the least trace present in the samples we examined, or because it was not possible to disclose it by the methods which we employed.

We accept the similarity which it has been sought to establish between the salivary concretions and the urinary calculus. The etiology is the same, and in the one case as in the other we have before us a microbian process. It is however, pushing the resemblance too far to say that the uric acid is to be found in all the concretions.

The chemical composition of salivary tartar closely approaches to these calcareous concretions, which are found in all the osseus parts of the body and in the bladder, every time that a process of the purely parasitical order comes into action.

It seems to us that the exclusively gouty origin of pyorrhea alveolaris ought not to be accepted, although it may have been established for a long time that the arthritics and rheumatics offer a predisposed field for the development of this disease, in which, as we have demonstrated with M. Malassez, the infectious element plays such a preponderating part.

After a decade the infectious character attributed by us to pyorrhea alveolaris comes back to us from America as a discovery, without the least mention being made of the researches of M. Malassez and my own. This is too much in the order of things for us to think of complaining.—*Journal des Connaissances Medicales*, quoted in *L'Odontologie.*

